# Identification of Novel Genes in Osteoarthritic Fibroblast-Like Synoviocytes Using Next-Generation Sequencing and Bioinformatics Approaches

**DOI:** 10.7150/ijms.35611

**Published:** 2019-07-21

**Authors:** Yi-Jen Chen, Wei-An Chang, Ling-Yu Wu, Ching-Fen Huang, Chia-Hsin Chen, Po-Lin Kuo

**Affiliations:** 1 Graduate Institute of Clinical Medicine, College of Medicine, Kaohsiung Medical University, Kaohsiung 807, Taiwan; 2 Department of Physical Medicine and Rehabilitation, Kaohsiung Medical University Hospital, Kaohsiung 807, Taiwan; 3 Division of Pulmonary and Critical Care Medicine, Kaohsiung Medical University Hospital, Kaohsiung 807, Taiwan; 4 Department of Physical Medicine and Rehabilitation, School of Medicine, College of Medicine, Kaohsiung Medical University, Kaohsiung 807, Taiwan; 5 Orthopaedic Research Center, Kaohsiung Medical University, Kaohsiung 807, Taiwan; 6 Center for Cancer Research, Kaohsiung Medical University

**Keywords:** osteoarthritis, synovitis, fibroblast-like synoviocytes, next-generation sequencing, messenger RNA, microRNA, bioinformatics

## Abstract

Synovitis in osteoarthritis (OA) the consequence of low grade inflammatory process caused by cartilage breakdown products that stimulated the production of pro-inflammatory mediators by fibroblast-like synoviocytes (FLS). FLS participate in joint homeostasis and low grade inflammation in the joint microenvironment triggers FLS transformation. In the current study, we aimed to identify differentially expressed genes and potential miRNA regulations in human OA FLS through deep sequencing and bioinformatics approaches. The 245 differentially expressed genes in OA FLS were identified, and pathway analysis using various bioinformatics databases indicated their enrichment in functions related to altered extracellular matrix organization, cell adhesion and cellular movement. Moreover, among the 14 dysregulated genes with potential miRNA regulations identified, src kinase associated phosphoprotein 2 (*SKAP2*), adaptor related protein complex 1 sigma 2 subunit (*AP1S2*), PHD finger protein 21A (*PHF21A*), lipoma preferred partner (*LPP*), and transcription factor AP-2 alpha (*TFAP2A*) showed similar expression patterns in OA FLS and OA synovial tissue datasets in Gene Expression Omnibus database. Ingenuity Pathway Analysis identified the dysregulated *LPP* participated in cell migration and cell spreading of OA FLS, which was potentially regulated by miR-141-3p. The current findings suggested new perspectives into understanding the novel molecular signatures of FLS involved in the pathogenesis of OA, which may be potential therapeutic targets.

## Introduction

Osteoarthritis (OA) is one of the common articular disorders that affect major weight bearing joints, causing joint pain and stiffness and lead to chronic disability 1. The increasing prevalence of OA is likely due to increases in longevity and prevalence of obesity 2,3. Clinically, the diagnosis of OA is mainly based on symptoms and radiographic findings, although discordance between pain and severity of radiographic joint pathology has been reported 4,5. The major histopathological changes in OA joint are the cartilage destruction with hypertrophic differentiation of chondrocytes 6. However, the contribution of low-grade inflammation and synovitis in OA progression has been appreciated, and OA is now considered a disease of the whole joint, not merely the cartilage 7,8.

The synovium forms the boundary between internal joint structure and adjacent soft tissues, and is essential for maintaining joint homeostasis. The major cellular components of this distinct tissue layer are the fibroblast-like and macrophage-like synoviocytes. The fibroblast-like synoviocytes (FLS) produce major constituents of synovial fluid that nourishes the chondrocytes through synovial vascular network, while the synovial macrophages help clearing debris from minor joint injuries 9. Synovitis is a common feature of inflammatory arthritis, including rheumatoid arthritis (RA) and OA, and the degree of synovitis is associated with joint pain and structural progression 7,10. The low-grade inflammatory OA joint microenvironment is caused by the cartilage breakdown products that provoke the release of proteolytic enzymes and increased production of pro-inflammatory mediators from FLS, followed by immune cell infiltration and vascular hyperplasia, leading to synovial inflammation 7,8. This synovial change and overexpression of pro-inflammatory mediators can be observed in the early stage of OA, even before the presence of macroscopic cartilage degeneration 11.

OA being a disease of the whole joint involving the cartilage, synovium and subchondral bone, and synovitis associated with symptoms and progression of OA, the synovium may serve as a potential therapeutic target in the management of OA 8. While several studies focusing on OA synovial fluid and FLS have proposed the role of microRNAs (miRNAs) in the pathogenesis of OA synovitis and disease progression 12-14, novel therapeutics targeting these small non-coding single-stranded RNAs through intra-articular injection may contribute to the maintenance of joint homeostasis, fine tuning downstream gene expressions related to inflammatory and catabolic pathways 14-16. The transcriptome changes and novel molecular signatures between normal and arthritic pathologies can be efficiently identified using the high-throughput next-generation sequencing (NGS) technique 17,18, and the biological themes underlying the differentially expressed genomic profiling can be determined through the integrated analysis with bioinformatics approaches 19-21.

In the current study, the biological functions underlying the differentially expressed genes and potential miRNA regulations in OA FLS will be investigated using NGS and different bioinformatics databases, and validated in clinical OA synovium tissue data available in functional genomics data repository. We propose the findings will gain novel insights into understanding the role of FLS in the pathogenesis of OA and identify potential therapeutic targets in the management of OA.

## Materials and Methods

### Culturing Human Fibroblast-Like Synoviocytes (HFLS)

Human fibroblast-like synoviocytes isolated from adult normal (HFLS) and osteoarthritic synovial tissue (HFLS-OA) were obtained from Cell Applications, Inc. (San Diego, CA, USA). The isolated cells were cryopreserved at the first passage. The cryopreserved vials of HFLS and HFLS-OA were thawed and cultured in Synoviocyte Growth Medium (Cell Applications, Inc. San Diego, CA, USA) and incubated in a 37°C, 5% CO_2_ humidified incubator until confluence. The cells were then harvested for total RNA extraction using Trizol Reagent (Invitrogen, Carlsbad, CA, USA). The quality of extracted RNAs were confirmed using ND-1000 spectrophotometer (Nanodrop Technology, Wilmington, DE, USA) for detection of OD_260_/OD_280_ absorbance ratio and Bioanalyzer 2100 (Agilent Technology, Santa Clara, CA, USA) for RNA integrity number (RIN) with RNA 6000 labchip kit (Agilent Technology, Santa Clara, CA, USA). The OD_260_/OD_280_ absorbance ratio was 1.95 for HFLS and 1.94 for HFLS-OA, while the RINs were 9.9 and 10 for HFLS and HFLS-OA, respectively, indicating good quality of the extracted RNA.

### RNA Sequencing

The RNA and small RNA sequencing were carried out by Welgene Biotechnology Company (Welgene, Taipei, Taiwan). For RNA sequencing, all RNA samples were prepared according to the Illumina protocol. The Agilent's SureSelect Strand Specific RNA Library Preparation Kit was used for RNA library construction, followed by AMPure XP Beads size selection. The sequence was determined by sequencing-by-synthesis technology, with read length at 150 nucleotides pair-end. The sequence data was generated by Welgene's pipeline based on Illumina bcl2fastq v2.1.7. The raw reads were trimmed for qualified reads and remove lower quality bases using Trimmomatic (version 0.32), and the qualified reads were then aligned to reference human genome using HISAT2 alignment tool. The expression level of each aligned gene was normalized and expressed in fragments per kilobase of transcript per million mapped reads (FPKM). The differential expression between HFLS and HFLS-OA were analyzed based on Cuffdiff (Cufflinks version 2.2.1) with genome bias detection/correction and Welgene in-house programs. For small RNA sequencing, samples were prepared using Illumina sample preparation kit following the TruSeq Small RNA Sample Preparation Guide. The RNAs were reversed transcribed to cDNA, size-fractionated and purified to obtain bands with 18-40 nucleotides. The sequencing with read length at 75 nucleotides single-end was carried out on Illumina instrument and processed with Illumina software. The raw reads were trimmed for qualified reads and analyzed using miRDeep2 to clip 3' adaptor sequence before aligning to reference human genome from University of California, Santa Cruz (UCSC). The expression levels of known miRNAs were estimated using miRDeep2, normalized in reads per million (RPM). The selection criteria for differentially expressed mRNAs and miRNAs between HFLS and HFLS-OA were as following: fold change > 2.0, FPKM > 0.3 for mRNA and RPM > 1 for miRNA in at least one group.

### Functional Enrichment Analysis Using Different Bioinformatics Tools

The gene lists of interest were uploaded into Database for Annotation, Visualization and Integrated Discovery (DAVID) bioinformatics resource 22 and Ingenuity Pathway Analysis (IPA) software (Ingenuity systems, Redwood City, CA, USA) 23 to perform integrated data mining and categorize large gene lists into different enriched biological functions and/or networks. The IPA software was also able to predict potential upstream regulators and downstream effectors of a given gene list. In the DAVID database, differentially expressed genes were uploaded for functional annotation analysis, setting the Expression Analysis Systematic Explorer (EASE) score at default cutoff value of 0.1, which represented the modified Fisher's exact p-value. In the IPA software, differentially expressed genes with fold changes between HFLS and HFLS-OA were uploaded for core analysis. The analytic results were obtained based on all direct and indirect relationships identified in all tissue types, and from either experimentally observed or moderate to highly predicted confidence.

### Protein-Protein Interaction Network Analysis Using STRING Database

To identify the protein-protein interaction (PPI) network of differentially expressed genes, the STRING database (version 11.0) integrating functional interactions from known and predicted protein-protein association data was used 24. For sub-network analysis, the Molecular Complex Detection (MCODE) plugin tool under Cytoscape software package was used to cluster the large PPI network into small networks 25.

### MiRNA Target Prediction

For those identified differentially expressed miRNAs between HFLS and HFLS-OA, the putative targets were predicted using the miRmap database (miRmap version 1.0), an open-source software library that was developed using a comprehensive approach to predict the repression strength of a miRNA to specific genes 26. Higher miRmap scores indicated higher repression strength. In the current study, 83 differentially expressed miRNAs were analyzed for their putative targets, and those putative targets with miRmap scores higher than 99.0 were selected. In addition, those potential miRNA-mRNA interactions of interest were further validated in other two miRNA prediction databases, including TargetScan 27 and miRDB 28.

### Functional Genomics Data Repository -- Gene Expression Omnibus (GEO) Database

To assess the expression patterns of candidate genes of interest in clinical OA synovial tissue samples, we searched in the GEO database 29 for related high-throughput genomic datasets on synovial tissues from normal and OA patients. The genes of interest with their expression values could be obtained for further between-group comparison. In the current study, we assessed the expression patterns of candidate genes in five datasets of normal and OA synovial tissue samples (GSE55235, GSE55457, GSE82107, GSE1919 and GSE29746) and one dataset comparing non-inflammatory and inflammatory OA synovial tissues (GSE46750).

### Statistical Analysis

The between-group difference of target gene expression values identified from selected GEO datasets were analyzed using non-parametric Mann-Whitney *U* test with SPSS Statistics software (version 19, IBM Corp., Armonk, NY, USA). A p-value < 0.05 was considered statistically significant.

## Results

### Identification of Differential Expression Profile between Normal and Osteoarthritic Human Fibroblast-Like Synoviocytes

**Figure 1:**
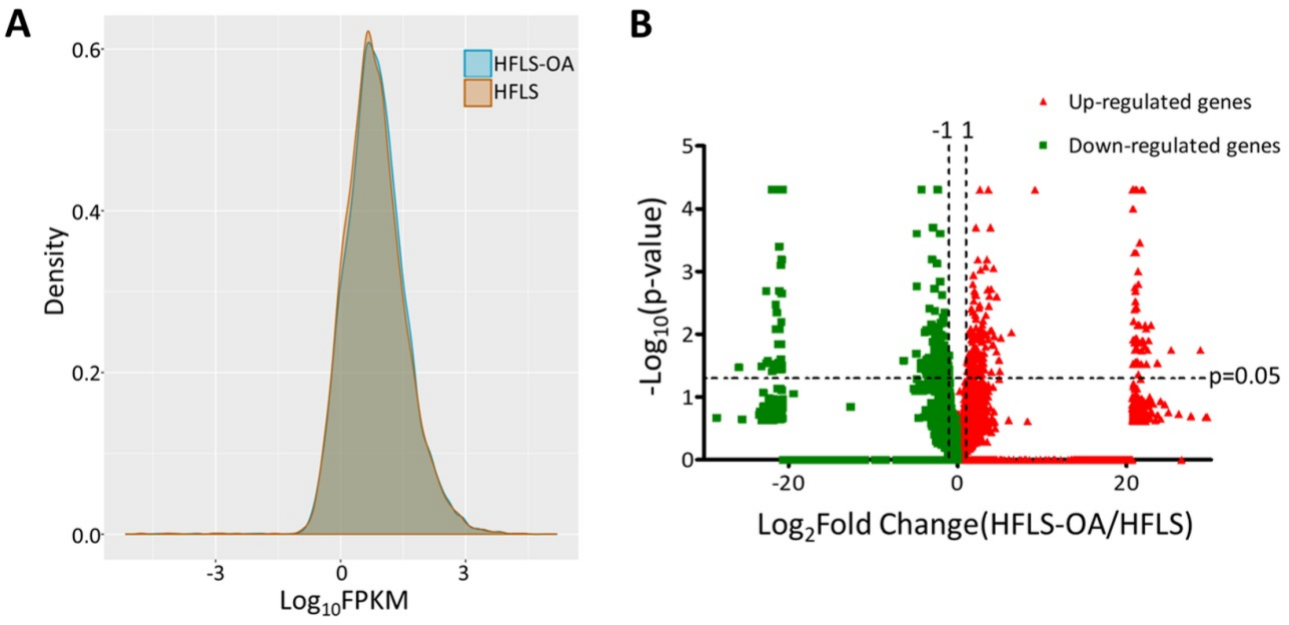
(A) The gene expression from next-generation sequencing in fragments per kilobase of transcript per million mapped reads (FPKM) performance of normal (HFLS) and osteoarthritic (HFLS-OA) human fibroblast-like synoviocytes were displayed in density plot. (B) The differential expression patterns between HFLS and HFLS-OA were plotted in volcano plot. The red dots represented up-regulated genes and the green dots represented down-regulated genes in HFLS-OA. Those genes with fold changes > 2.0 and p value < 0.05 were selected as significantly dysregulated genes.

The transcriptomic profile of adult HFLS and HFLS-OA cells were obtained from NGS results and the FPKM performance between two samples were displayed in density plot, as shown in Figure 1A. The differentially expressed genes between HFLS and HFLS-OA were screened for according to the following selection criteria: expression higher than 0.3 FPKM in either sample, at least two-fold change between HFLS and HFLS-OA, and significant differential expression with p-value < 0.05. The distribution of differential expression genes between HFLS and HFLS-OA were displayed in volcano plot (Figure 1B). The selection criteria yielded a total of 118 significantly up-regulated genes and 127 significantly down-regulated genes in HFLS-OA cells.

### The Differentially Expressed Genes were Enriched in Functions Related to Extracellular Matrix Organization, Cell Adhesion and Cellular Movement

**Figure 2:**
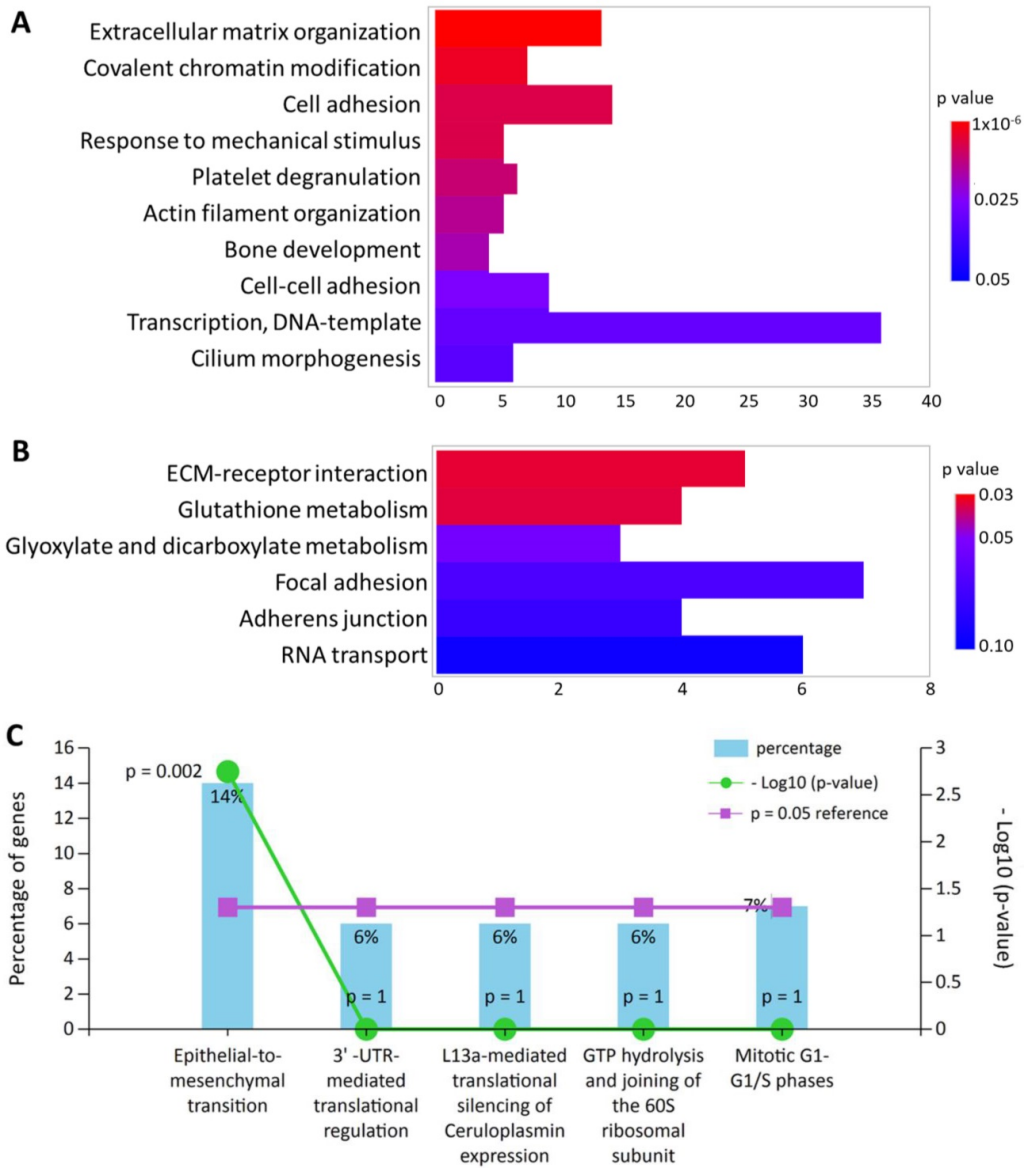
The top enriched (A) biological processes in Gene Ontology terms and (B) Kyoto Encyclopedia of Genes and Genomes pathways in differentially expressed genes of HFLS-OA were identified from the Database for Annotation, Visualization and Integrated Discovery bioinformatics resource. The color scale indicated the corresponding p values and the x-axis indicated the gene counts of each biological function. (C) The enriched biological pathway in differentially expressed genes of HFLS-OA were identified from the FunRich database, where the percentage of genes and -log(p-value) of each biological pathway were indicated.

All 245 differentially expressed genes were uploaded into DAVID database for terms of biological process in Gene Ontology and Kyoto Encyclopedia of Genes and Genomes (KEGG) pathway. In addition, these differentially expressed genes were also input into FunRich database for functional enrichment analysis. The functionally enriched biological processes, KEGG pathways and biological pathways with their p-values were shown in Figure 2. The top enriched functions were related to extracellular matrix (ECM) organization (p = 9.92x10^-6^) and cellular movement such cell adhesion (p = 0.007) and epithelial-to-mesenchymal transition (p = 0.002). Five genes were also found to be associated with “response to mechanical stimulus” from the DAVID database (p = 0.007), including *COL3A1*, *CHI3L1*, *POSTN*, *ASNS* and *CITED2*, which were all down-regulated in HFLS-OA. Moreover, genes with differential expression values and fold-changes were also uploaded into IPA for core analysis. The results showed “cellular movement” was the top enriched molecular and cellular function, with 29 related molecules involved. Besides, the function annotation of “cell spreading” (p = 0.00238, z-score = -2.121) was predicted to have decreased activation, with the following molecules involved: *CAP1*, *CDH11*, *LPP*, *MYH10*, *SERPINE1*, *SMAD4*, *SPARC*, *TGFBI*.

### Identification of Enriched Functions in Differentially Expressed Gene Clusters of HFLS

**Figure 3:**
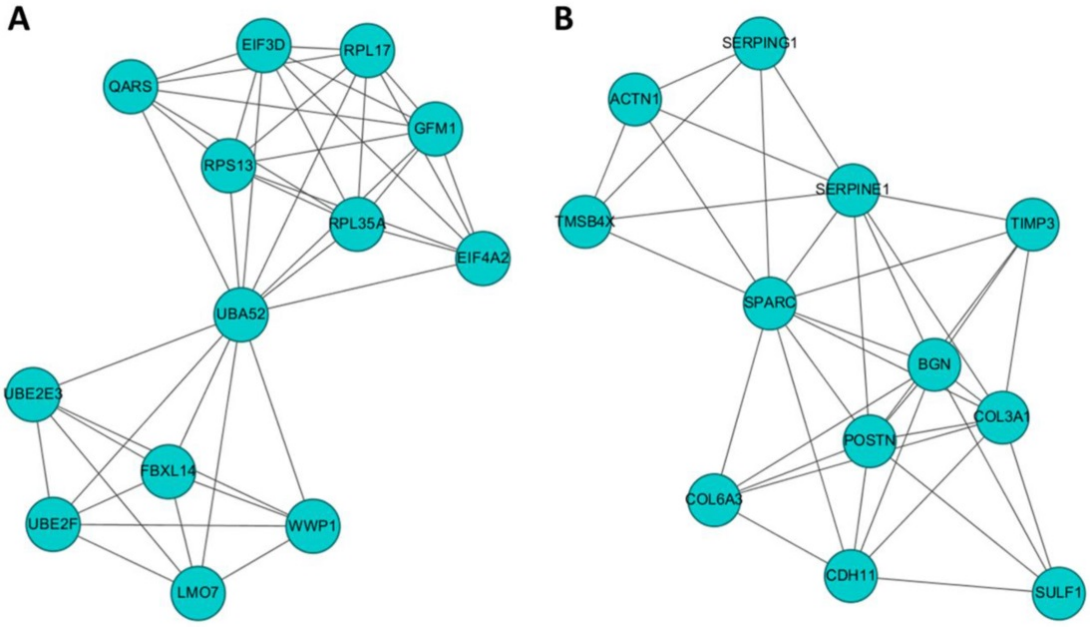
The potential interaction networks of (A) cluster 1 containing 13 molecules and (B) cluster 2 containing 12 molecules identified from Molecular Complex Detection (MCODE) were indicated. The two sub-networks were drawn from the Cytoscape software.

To identify gene clusters among the 245 differentially expressed genes in HFLS-OA and their associated biological functions, the list of differentially expressed genes were input into the STRING database to obtain a large PPI network. The sub-cluster analysis was performed under the Cytoscape software with plug-in tool MCODE. The sub-clusters of networks from MCODE were listed in Table 1, with cluster 1 and cluster 2 having higher scores. The two clusters of sub-networks were drawn in the Cytoscape software, as shown in Figure 3. To understand the biological functions of these two clusters of genes, the two clusters were separately input into DAVID database for functional annotation analysis. The top enriched biological functions in terms of biological process and KEGG pathway were listed in Table 2. Genes in cluster 1 were associated with RNA and protein processing, while genes in cluster 2 were associated with ECM organization and cell focal adhesion.

### Identification of Differentially Expressed miRNAs and Potential miRNA-mRNA Interactions in HFLS-OA Cells

**Figure 4:**
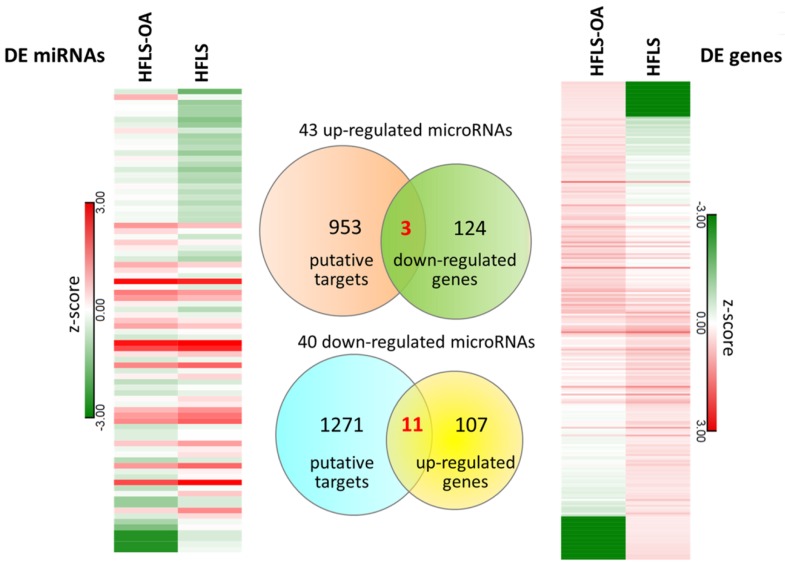
The differentially expressed miRNAs and mRNAs in HFLS and HFLS-OA displayed in heatmaps were indicated in left and right panels, respectively. Putative targets of dysregulated miRNAs were predicted from the miRmap database, selecting those with miRmap scores of ≥ 99.0 indicating high repression strength. The putative targets were matched to differentially expressed mRNAs in HFLS, and the Venn diagram was displayed in the middle panel. A total of 11 up-regulated genes and 3 down-regulated genes with potential miRNA regulations were identified.

The differential miRNA expression profile between HFLS and HFLS-OA were simultaneously investigated with small RNA sequencing. The selection criteria for differentially expressed miRNAs in HFLS-OA were as following: normalized read counts > 1 RPM, at least 2.0-fold-change between HFLS and HFLS-OA. The result yielded 43 up-regulated and 40 down-regulated miRNAs in HFLS-OA. To obtain putative targets of dysregulated miRNAs, miRmap database, a miRNA target prediction database, was used, and those predicted targets with miRmap scores of at least 99.0 were selected. There were 956 putative targets of 43 up-regulated miRNAs and 1282 putative targets of 40 down-regulated miRNAs identified. These putative targets of up- and down-regulated miRNAs were matched to our differential expression mRNA profiles of 127 down- and 118 up-regulated genes in HFLS-OA. The heatmaps of differentially expressed miRNAs and mRNAs in z-score and the Venn diagram were shown in Figure 4. A total of 14 target genes with potential miRNA regulations were selected. The detailed gene names and their expression values in FPKM were listed in Table 3.

### Analysis of Target Genes with Potential miRNA-mRNA Interactions in Osteoarthritic Synovial Tissues and Identification of Potential Molecular Signatures in Osteoarthritic Synovium

**Figure 5:**
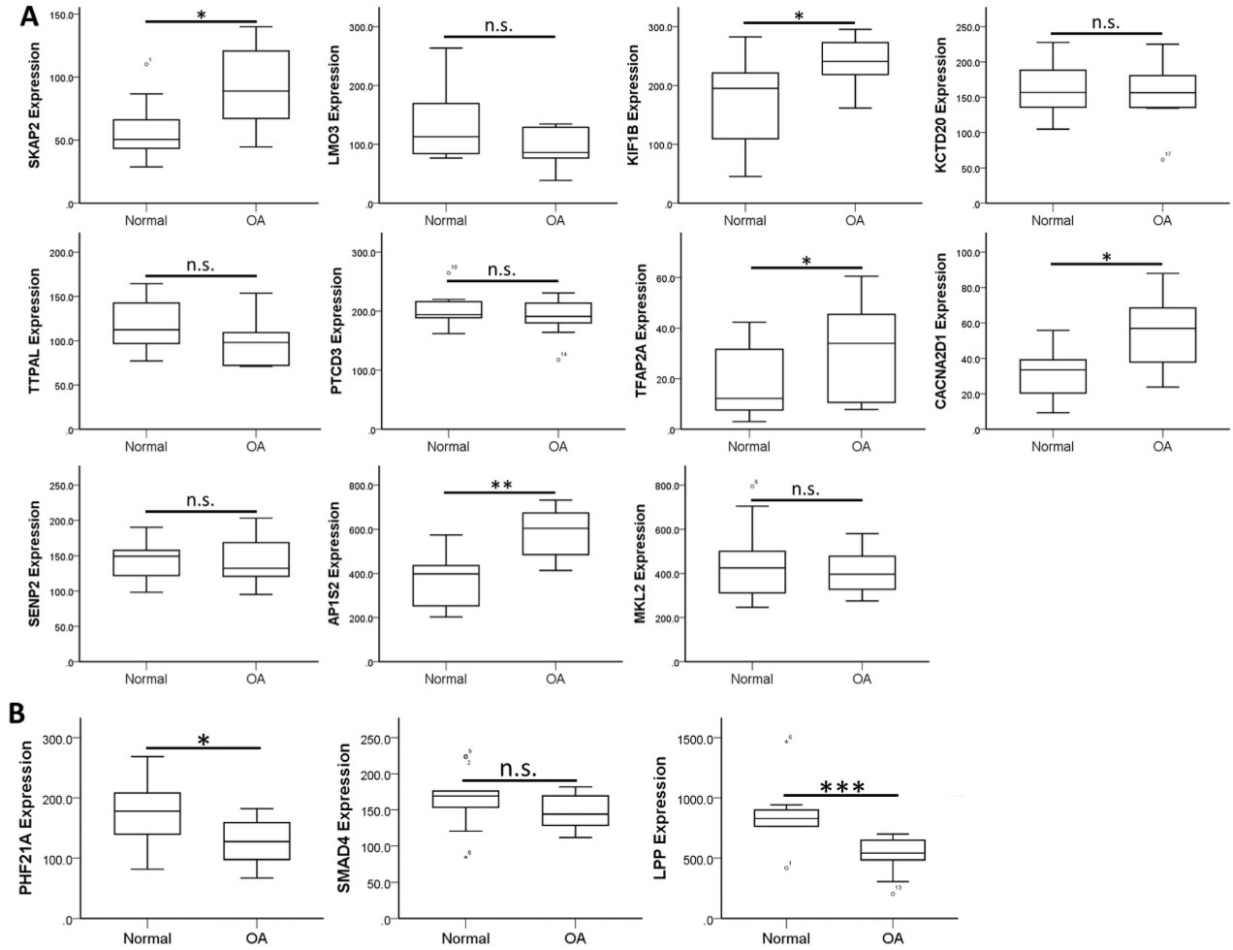
The expression patterns of 14 target genes, including (A) 11 up-regulated and (B) 3 down-regulated genes were analyzed in one of the OA synovial tissue datasets extracted from GEO database (GSE55235). The significantly up-regulated expressions in *SKAP2*, *KIF1B*, *TFAP2A*, *CACNA2D1* and *AP1S2*, and significantly down-regulated expressions in *PHF21A* and *LPP* in OA knee synovial tissues were in similar expression patterns to our HFLS-OA data. * indicated p < 0.05, ** indicated p < 0.01, *** indicated p < 0.001, and n.s. indicated no statistical significance between normal and OA groups.

To validate the expression patterns of these 14 target genes in clinical OA synovial tissues, we searched in the GEO database for OA synovial tissue datasets to further analysis of the expression patterns. Those datasets containing both normal and OA synovial tissue samples were selected for expression analysis. There were four OA synovial tissue datasets (GSE55235, GSE55457, GSE82107 and GSE1919) and one OA synovial fibroblast dataset (GSE29746) found in the database. In addition, one dataset comparing non-inflammatory and inflammatory OA synovial tissue expression profile (GSE46750) was also selected for analysis. The expression levels of the 14 target genes were analyzed in these 6 datasets to search for similar expression patterns found in our HFLS-OA data. The expression patterns of these target genes in the 6 datasets were summarized in Table 4. The more consistently dysregulated expression patterns in *SKAP2*, *AP1S2*, *PHF21A* and *LPP* were found in our HFLS-OA dataset and in at least two of the four OA synovial tissue datasets. Moreover, the down-regulated *LPP* was also observed in synovial fibroblast dataset. Additionally, the up-regulated *TFAP2A* was also found up-regulated in inflammatory OA synovial tissue samples. The expression patterns of the 14 target genes in one of the representative datasets (GSE55235) was shown in Figure 5.

### Determination of Potential miRNA-mRNA Interactions in HFLS-OA

Since the expression patterns of *SKAP2*, *AP1S2*, *PHF21A*, *LPP*, and *TFAP2A* were more consistently observed in our HFLS-OA NGS dataset and OA synovial tissue datasets from GEO database, we further analyzed in the miRmap database for potential miRNA regulations of these candidate genes. Those potential miRNA-mRNA interactions with miRmap scores higher than 99.0 were selected and matched to our HFLS dataset of differentially expressed miRNAs. A total of 11 potential miRNA-mRNA interactions were identified. We then validated the putative 3'UTR binding sites and sequences of these potential miRNA-mRNA interactions in TargetScan and miRDB miRNA prediction databases. The results were listed in Table 5, and there were four potential miRNA regulations consistently validated in all three miRNA target prediction databases, including hsa-miR-450b-5p-*SKAP2*, hsa-miR-204-5p-*AP1S2*, hsa-miR-766-3p-*PHF21A* and hsa-miR-141-3p-*LPP*.

**Figure 6:**
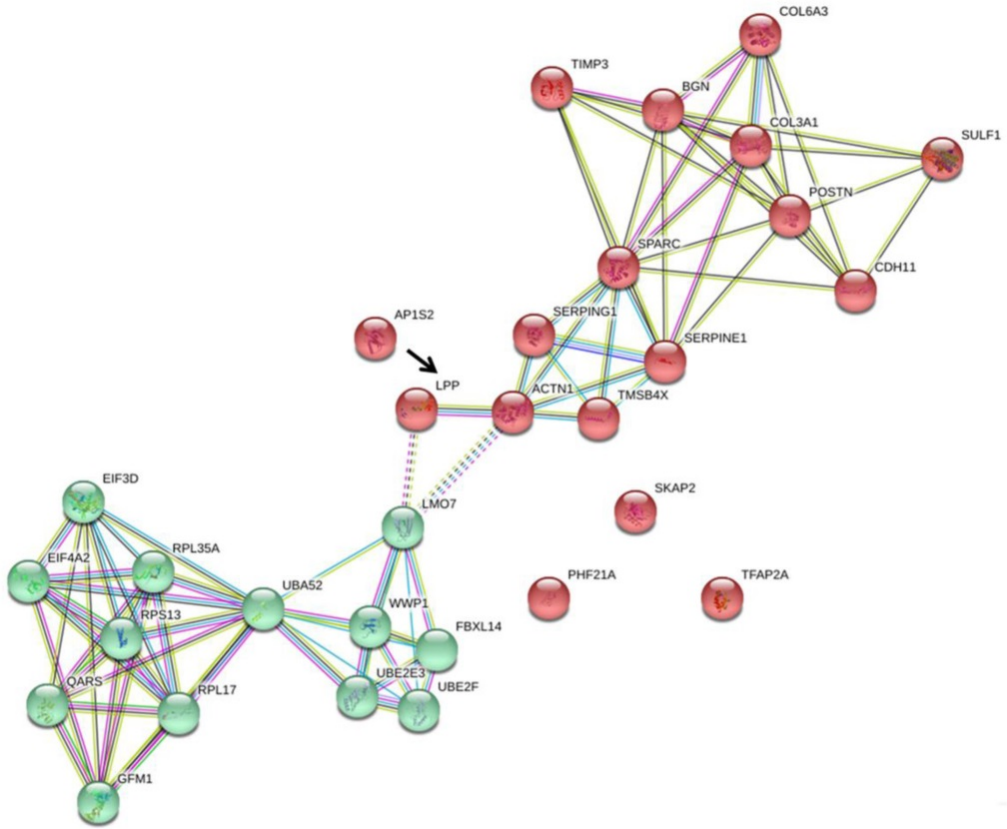
The five putative targets with potential miRNA regulations, including *SKAP2*, *AP1S2*, *PHF21A*, *LPP*, and *TFAP2A*, along with the two clusters of genes previously identified were input into STRING database for potential interaction network. Among the 5 putative targets, we found that *LPP* (indicated in black arrow) was the only molecule having direct interaction with *ACTN1* in cluster 2, and indirect interaction with *LMO7* in cluster 1.

To understand the association of these miRNA targets among the two main clusters of differentially expressed genes in HFLS-OA, we uploaded these five target genes, *SKAP2*, *AP1S2*, *PHF21A*, *LPP*, and *TFAP2A* along with the two clusters of genes into the STRING database for interaction network analysis. The interaction network drawn from the STRING database was shown in Figure 6, where *LPP* was the only molecule having close association with *ACTN1*, one of the molecules in cluster 2.

### LPP was Potentially Associated with Cell Migration and Cell Spreading

**Figure 7:**
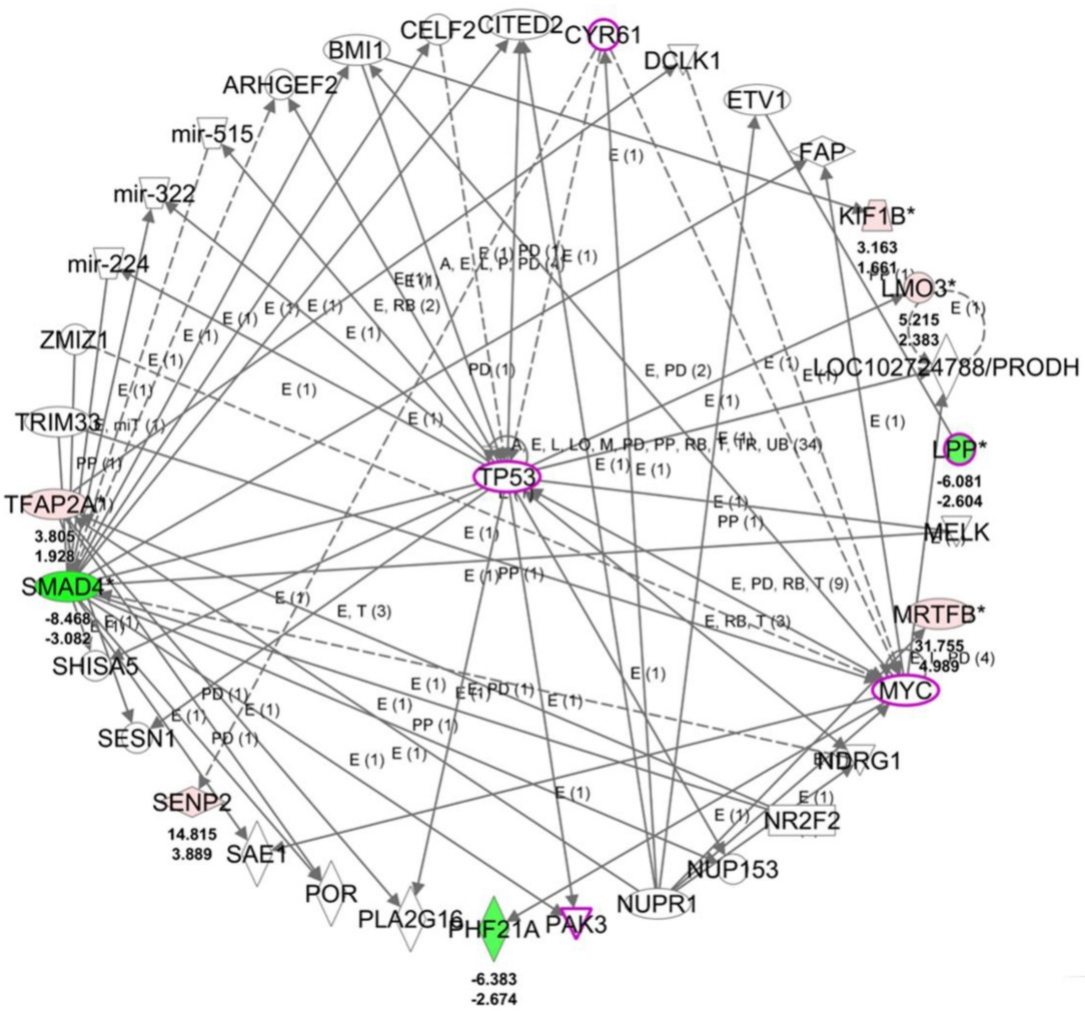
The top scored network of the 14 target genes identified from the IPA software indicated 8 of the 14 genes were group in this network related to cellular development, cellular growth and proliferation, and neurological disease. The overlay diseases and functions analysis indicated *TP53*, *PAK3*, *MYC*, *LPP* and *CYR61* (marked in purple frames) were associated with migration of fibroblasts. Molecules in red indicated up-regulated expression and molecules in green indicated down-regulated expression in HFLS-OA data. The color scales indicated the relative expression values of HFLS-OA to HFLS. The numbers indicated below each colored molecule indicated fold-changes and log_2_(ratio) of HFLS-OA to HFLS expression.

To understand the potential biological themes among the 14 target genes with potential miRNA regulations, these target genes were uploaded to the IPA software for functional enrichment analysis. The top networks from the IPA core analysis result indicated 8 of the 14 target genes were clustered in the top scored network related to diseases and functions of cellular development, cellular growth and proliferation, and neurological disease, in which *LPP* was one of the molecules in this top network. The detailed networks of these target genes were listed in Table 6. The interactions between the molecules in network 1 was shown in Figure 7, where tumor protein 53 (*TP53*) was the central hub of the network. Further overlay diseases and functions analysis indicated *TP53*, *PAK3*,* MYC*, *LPP*, and *CYR61* (marked in purple frame in Figure 7) were associated with “migration of fibroblasts”. Along with previous finding from IPA that *LPP* was one of the molecules predicted to be involved in cell spreading, it is suggested that *LPP* potentially regulated by miR-141-3p was involved in functions of cell migration and cell spreading.

## Discussion

**Figure 8:**
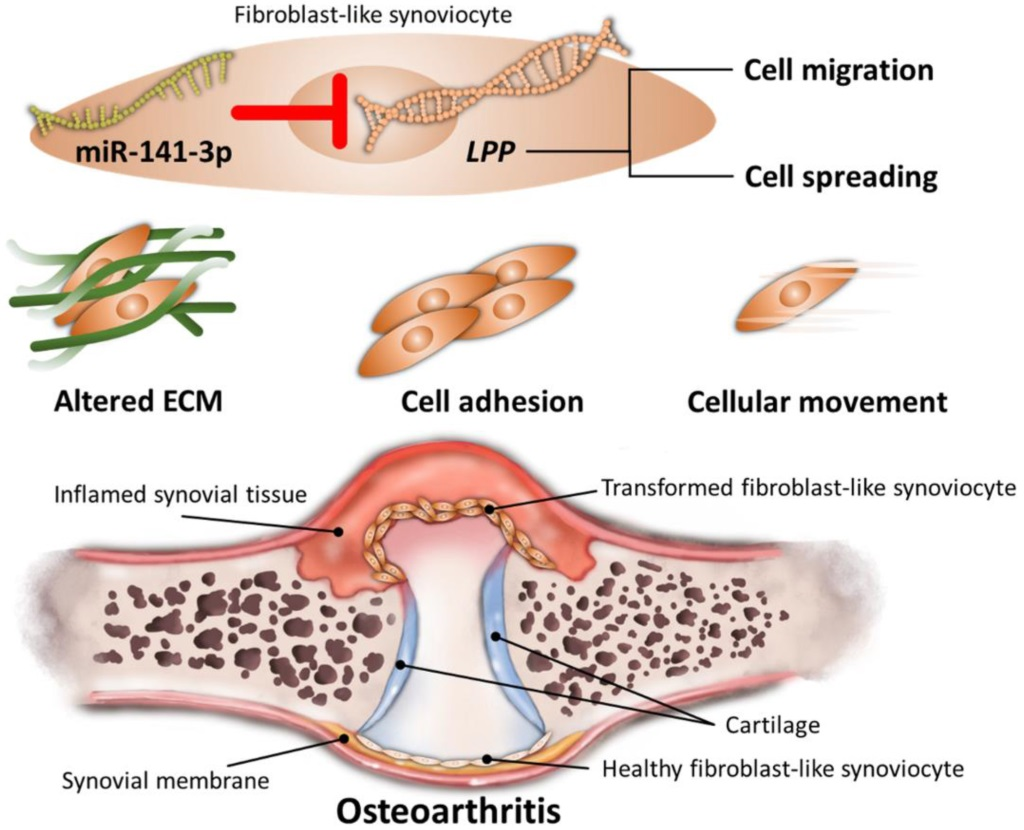
Schematic summary of novel molecular signatures in OA fibroblast-like synoviocytes

Through NGS and bioinformatics analysis, our current study identified the differentially expressed genes in HFLS-OA were enriched in functions related to ECM organization, cell adhesion and cellular movement. Moreover, the dysregulated *LPP* was suggested to participate in altered cellular movement of OA synoviocytes, potentially regulated by miR-141-3p, systematically validated through different miRNA prediction databases. The schematic summary of the novel molecular signatures identified in our current study is shown in Figure 8.

Synovitis is one of the macroscopic structural changes of OA, affecting joint integrity 30. FLS transformation and synovitis have been reported to contribute to inflammatory arthritis 9,31. In response to inflammation, FLS transformation occurs through activation of several signaling pathways, for instance, toll-like receptor signaling and epigenetic control 9. Transformed FLS become aggressive in behavior and lead to persistent synovitis; they can proliferate rapidly and are highly migratory, and attach to the articular cartilage and produce matrix degrading enzymes to degrade and invade the cartilage 9. The aggressive behavior of FLS in synovitis of RA and OA may differ in extend. In RA, diffuse synovial inflammation is observed, while synovitis in OA is usually in patchy distribution, confined to sites adjacent to cartilage damage 30. The increased migratory and invasive ability of FLS have been more extensively recognized in RA than in OA 9,32, and several transcription factors are reported to be potentially responsible for the FLS transformation in inflammatory arthritic diseases 33,34. In OA, several studies suggested that activation of receptors for advanced glycation end products and up-regulated expression of chemokine receptor 3 and cadherin-11 could increase the catabolic activity and migratory/invasive capacity of FLS 35-37. In our HFLS-OA data, bioinformatics analysis suggested top enriched functions related to ECM organization and altered cellular movement, while the IPA results predicted the decreased activation in the cellular function of “cell spreading” (p = 0.00238, z-score = -2.121). The aggressiveness of transformed FLS may be affected by the severity of inflammatory arthritis 30,36. The complexity of cell migration in synovial joint lies in the multifaceted biological factors dependent on cellular and ECM properties 38. The location of synovial tissue in the affected OA joint may vary in histopathological changes 39,40, and the severity and degree of synovitis in OA may also influence the expression levels of cell adhesion and cellular movement related genes in FLS 41. Of the 8 genes associated with function of cell spreading, including *CDH11*, *LPP*,* SERPINE1*, *SMAD4*, *SPARC*, *TGFBI, CAP1*, and *MYH10*, the dysregulated *LPP* is the candidate gene deducted from the differentially expressed mRNA results.

Through systematic bioinformatics analysis, we deducted the novel gene, *LPP*, 6.08-fold down-regulated in HFLS-OA potentially participated in altered cellular movement of OA synoviocytes. *LPP* (lipoma preferred partner) gene encodes LPP protein, a member of the zyxin family of LIM domain proteins that localizes at sites of cell adhesion and cell-cell contacts 42,43. Besides, it also interacts with α-actinin (*ACTN1*) to participate in diverse cellular processes such as cell adhesion, spreading and migration 44. *LPP* may partner with different molecules and reflect its various functions, therefore,* LPP* may exert as potential oncogene or oncosuppressor gene in different cancer cell lines 45,46. The role of *LPP* in arthritic joint tissues has not been reported. However, *ACTN1* was demonstrated to have increased expression in synovial tissues of RA compared to OA, and may participate in the signaling pathway initiated by TNF-α, thereby inducing cell proliferation and spreading of RA fibroblast-like synoviocytes to unaffected joints 47,48. In our current study, OA FLS were obtained from commercialized human OA primary FLS. Whether the expression level of *LPP* can represent graded changes in different stages of OA and degree of synovitis and its interaction at sites of focal adhesion in OA FLS merits future investigation and validation in clinical synovial tissues of different OA stage subgroups.

The 2.37-fold up-regulated miR-141-3p in HLFS-OA was the miRNA potentially regulating *LPP* through systematic validation. The role of miR-141-3p in arthritis is still less investigated. One recent study by Park *et al*. reported the dysregulated miR-141-3p through ATP‐binding cassette transporter in human OA chondrocytes participated in altered lipid metabolism and suggested a novel insight into understanding the pathogenesis of OA 49. In view of inflammatory conditions, miR-141 seemed to play dual roles in regulating inflammation-related signaling pathways. In diet-induced steatohepatitis mice, level of miR-141 in liver was significantly increased, and miR-141 knockout diminished hepatic inflammation and steatosis 50. However, a recent report suggested that miR-141 decreased LPS-induced inflammation in human fibroblasts through p38 MAPK and NF-κB pathways 51. As to the effect of miR-141-3p on cell function of fibroblasts, Feng *et al*. observed down-regulated miR-141-3p in keloid skin fibroblasts, and miR-141-3p exerted inhibitory effect on fibroblast proliferation and migration in keloids 52. The results may be in line with our current findings of up-regulated miR-141-3p in HFLS-OA and functionally enriched biological functions of cellular movement in differentially expressed genes of HFLS-OA, particularly the predicted decreased activation in cell spreading from the IPA results. An interesting finding from literature review showed that miR-141 triggers senescence of human fibroblasts through the regulation of *BMI1* expression 53. In our HFLS-OA dataset, we also found the significantly down-regulated *BMI1* in HFLS-OA, although it is not identified as one of the putative targets of miR-141-3p for its lower repression strength. Decreased autophagy in chondrocytes during aging leads to cellular senescence and ultimately OA progression 54. Synoviocytes also serve to maintain joint homeostasis; however less is discussed about the senescence of synoviocytes and its link to OA progression. Further investigation on the role of miR-141-3p in OA FLS may have its clinical significance.

*SKAP2* encodes src kinase associated phosphoprotein 2 and participates in different physiological processes, including integrin signaling, cell migration and cancer progression 55. Alenghat *et al*. reported that *SKAP2* is required for macrophage migration, chemotaxis and spreading, and may transmit signals required for cell migration 56, while others also claimed the importance of *SKAP2* in integrin activation and neutrophil recruitment 57. The evidence provides insight into further understanding of the essential role of* SKAP2* in inflammatory disorders, including arthritis. Additionally, *SKAP2* was detected in stromal cells infiltrating human gastric cancer tissues, and promoted tumor-associated macrophage infiltration and facilitated metastatic progression 58. In contrary, Shimamura *et al*. demonstrated knockdown of *SKAP2* in fibroblasts accelerated cell migration, and suggested the negative regulation of *SKAP2* on cell migration of fibroblasts and tumor invasion of glioblastoma cells 59. The evidence suggested that *SKAP2* may exert distinct regulatory effect in different cell types. Our NGS results of HFLS showed the expression of *SKAP2* in normal FLS was undetectable, while in OA FLS the expression of *SKAP2* was 19.76 FPKM. The role of *SKAP2* in OA FLS remains uncertain, but the expression pattern of* SKAP2* in OA synovium may be associated with degree of inflammation in the OA joint microenvironment, and we proposed higher expression of *SKAP2* may represent enhanced migratory effect of OA FLS that lead to synovial inflammation and predict cartilage breakdown in OA joint.

*AP1S2* is a gene encoding sigma 2 subunit of the adaptor protein complex 1, and mediates the assembly of clathrin and the recognition of sorting signals of membrane proteins 60. Studies related to *AP1S2* have mainly focused on its mutation in neurodevelopmental disorders 61-63. One recent report suggested miR-204, an effector of melanoma target therapy drug, exerts anti-migratory activity through targeting *AP1S2* 64. The potential miR-204-5p-*AP1S2* interaction has been observed in our HFLS-OA data, with a 9.4-fold increase in *AP1S2* expression and 3.22-fold decrease in miR-204-5p expression in HFLS-OA. To our understanding, there has been no related studies claiming the association of *AP1S2* with inflammatory arthritis; however, expression of miR-204 were found decreased in T cells of RA patients 65, and decreased expression of miR-204 may enhance cartilage tissue destruction among OA patients through targeting IL-1β 66. Whether miR-204-5p-*AP1S2* interaction participates in OA synovitis or transformation of OA FLS merits future investigation.

*PHF21A*, also known as *BHC80*, is the molecule that recognizes unmethylated H3K4, and essential for neuronal and craniofacial development, and *PHF21A* loss of function may result in dysregulation of histone methylation and drive neurodevelopmental disorders 67,68. In retinal endothelial cells, the expression of *PHF21A* was decreased under high glucose conditions, which was suggestive of the potential role in the development of diabetic retinopathy 69. In mouse fibroblasts, *PHF21A* was one of the nuclear cofactors that modulate proinflammatory cytokine target genes, implicating its regulatory role in inflammatory status 70. A 6.38-fold decrease in *PHF21A* in HFLS-OA was observed in our NGS result. *PHF21A* may be one of the potential inflammatory markers of OA, however, there is still lack of clinical or experimental evidence to link between *PHF21A* and OA synovitis in OA FLS.

Comparing the expression patterns of target genes with potential miRNA regulations identified from our HFLS-OA data in clinical samples of OA synovial tissues from GEO database, *TFAP2A* was the only gene significantly up-regulated in inflammatory OA synovium compared to that of non-inflammatory OA synovium. *TFAP2A* is a gene encoding activating enhancer-binding protein-2α (AP-2α) transcription factor, one of the members of AP-2 transcription factors known to play critical roles in cell cycle regulation and cell survival 71. Previous studies suggested induction of transcription factor AP-2 expression during acute inflammation of rat primary afferent neurons 72 and in response to inflammatory cytokines in human keratinocytes 73, which are evident of possible AP-2 regulation through complex cytokine system and initiation of inflammatory process. Similar results were also observed in human lung fibroblasts and bronchial epithelial cells, in which induction of NF-κB and AP-2 led to IL-8 production and subsequent neutrophil infiltration and inflammation during bacterial infection 74. Little is known about the role of AP-2 in arthritis. In OA chondrocytes, transfection of transcription factor AP-2ε led to increased expression of *CXCL1*, one of the chemokines inducing chondrocyte hypertrophy and apoptosis in OA 75. Two recent studies also suggested *TFAP2A* is potentially one of the novel transcription regulators in psoriatic skin 76 and RA synoviocytes 77 through sequencing and bioinformatics approach. Taken together, we proposed the up-regulated *TFAP2A* in OA synovial tissue may represent higher degree of inflammation in OA, and *TFAP2A* may be a novel target for anti-inflammatory therapy in arthritis.

## Conclusions

The current study identified differentially expressed genes in OA FLS were enriched in functions related to altered ECM, cell adhesion and cellular movement. In addition, miR-141-3p-*LPP* interaction potentially participated in cell migration and cell spreading of OA FLS, validated systematically in different miRNA target prediction databases. The current findings suggested novel insights into understanding the molecular signatures of FLS involved in the pathogenesis of OA, which may be potential therapeutic targets for the management of OA.

## Figures and Tables

**Table 1:** Ranked clusters of networks of OA fibroblast-like synoviocytes (FLS) analyzed by MCODE

Cluster	Score (Density*#Nodes)	Nodes	Edges	Node IDs
1	7	13	42	UBE2F, LMO7, EIF3D, RPL17, EIF4A2, UBE2E3, QARS, RPS13, WWP1, UBA52, RPL35A, GFM1, FBXL14
2	6.727	12	37	SULF1, TIMP3, CDH11, TMSB4X, SERPINE1, POSTN, SERPING1, SPARC, BGN, ACTN1, COL6A3, COL3A1
3	4	4	6	MRPS28, MTRF1L, MRPL52, PTCD3
4	3	3	3	ACSS2, PGD, MDH2
5	3	3	3	TCTN1, RPGR, ARL13B
6	3	3	3	CPSF1, LSM5, SART1
7	3	3	3	GATAD2A, HDAC7, PHF21A

**Table 2:** Enrichment analysis of top 2 clusters of sub-network analyzed from MCODE

Sub-network		Count	Genes	P value	Fold Enrichment
**Biological process**					
Cluster 1	Translational initiation	6	RPL17, RPL35A, EIF3D, EIF4A2, RPS13, UBA52	2.54x10^-8^	56.57
	SRP-dependent cotranslational protein targeting to membrane	4	RPL17, RPL35A, RPS13, UBA52	3.60x10^-5^	54.97
	Viral transcription	4	RPL17, RPL35A, RPS13, UBA52	6.08x10^-5^	46.13
	Nuclear-transcribed mRNA catabolic process, nonsense-mediated decay	4	RPL17, RPL35A, RPS13, UBA52	7.29x10^-5^	43.42
	rRNA processing	4	RPL17, RPL35A, RPS13, UBA52	4.12x10^-4^	24.14
Cluster 2	Platelet degranulation	6	SERPINE1, ACTN1, SERPING1, TMSB4X, SPARC, TIMP3	3.53x10^-9^	81.51
	Extracellular matrix organization	6	BGN, COL3A1, COL6A3, SERPINE1, POSTN, SPARC	8.99x10^-8^	42.84
	Negative regulation of endopeptidase activity	3	COL6A3, SERPINE1, SERPING1	0.003	34.69
	Skeletal system development	3	COL3A1, POSTN, CDH11	0.003	30.64
	Fibrinolysis	2	SERPINE1, SERPING1	0.014	133.27
**KEGG pathway**					
Cluster 1	Ribosome	4	RPL17, RPL35A, RPS13, UBA52	8.19x10^-4^	18.39
	Ubiquitin mediated proteolysis	3	UBE2E3, WWP1, UBE2F	0.016	13.69
Cluster 2	Focal adhesion	3	COL3A1, COL6A3, ACTN1	0.012	14.31
	Complement and coagulation cascades	2	SERPINE1, SERPING1	0.059	28.48
	ECM-receptor interaction	2	COL3A1, COL6A3	0.074	22.59
	Protein digestion and absorption	2	COL3A1, COL6A3	0.074	22.33
	Amoebiasis	2	COL3A1, ACTN1	0.089	18.54

**Table 3:** The 14 target genes of OA FLS with potential miRNA regulations

Gene Symbol	Gene Name	HFLS-OA FPKM	HFLS FPKM	Fold-Change (HFLS-OA/HFLS)
*SKAP2*	src kinase associated phosphoprotein 2	19.76	0.00	1975500.00
*LMO3*	LIM domain only 3	29.63	5.68	5.21
*KIF1B*	kinesin family member 1B	33.37	10.55	3.16
*KCTD20*	potassium channel tetramerization domain containing 20	22.34	6.36	3.51
*TTPAL*	alpha tocopherol transfer protein like	36.16	9.55	3.78
*PTCD3*	pentatricopeptide repeat domain 3	22.16	2.01	11.04
*TFAP2A*	transcription factor AP-2 alpha	43.17	11.35	3.81
*CACNA2D1*	calcium voltage-gated channel auxiliary subunit alpha2delta 1	18.94	0.63	30.00
*SENP2*	SUMO1/sentrin/SMT3 specific peptidase 2	18.78	1.27	14.81
*AP1S2*	adaptor related protein complex 1 sigma 2 subunit	33.42	3.56	9.40
*MKL2*	MKL1/myocardin like 2	19.40	0.61	31.75
*PHF21A*	plant homeodomain finger protein 21A	7.90	50.42	-6.38
*SMAD4*	SMAD family member 4	2.66	22.52	-8.47
*LPP*	Lipoma preferred partner	6.09	37.06	-6.08

**Table 4:** Analysis of 14 target gene expressions in OA synovium from Gene Expression Omnibus database

Accession #	GSE55235	GSE55457	GSE82107	GSE1919	GSE46750	GSE29746
	**Synovium tissue**					**Synovial fibroblast**
**Group**	Normal/OA	Normal/OA	Normal/OA	Normal/OA	Non-inflammatory /Inflammatory	Normal/OA
**Sample size**	10/10	10/10	7/10	5/5	12/12	11/11
**Up-regulated mRNA**						
***SKAP2***	up	up	down	n.s.	n.s.	n.s.
***LMO3***	n.s.	n.s.	n.s.	n.s.	n.s.	n.s.
***KIF1B***	up	n.s.	n.s.	n.s.	n.s.	n.s.
***KCTD20***	n.s.	down	up	n.s.	down	n.s.
***TTPAL***	n.s.	n.s.	n.s.	--	n.s.	down
***PTCD3***	n.s.	n.s.	n.s.	--	n.s.	n.s.
***TFAP2A***	up	n.s.	n.s.	n.s.	up	n.s.
***CACNA2D1***	up	n.s.	down	n.s.	n.s.	n.s.
***SENP2***	n.s.	n.s.	n.s.	--	n.s.	n.s.
***AP1S2***	up	up	n.s.	n.s.	n.s.	n.s.
***MKL2***	n.s.	n.s.	down	--	up	n.s.
**Down-regulated mRNA**						
***PHF21A***	down	down	n.s.	n.s.	n.s.	n.s.
***SMAD4***	n.s.	n.s.	n.s.	n.s.	n.s.	n.s.
***LPP***	down	down	n.s.	down	n.s.	down

up, significantly up-regulated in OA (p<0.05); down, significantly down-regulated in OA (p<0.05); n.s., non-significant between normal and OA synovium. -- indicated no identical probes within the dataset.

**Table 5:** Potential miRNA regulations of putative targets in OA FLS

Putative mRNA	mRNA Fold Change (HFLS-OA/HFLS)	Predicted miRNA	miRNA Fold Change (HFLS-OA/HFLS)	miRmap Score	TargetScan	miRDB
*SKAP2*	1975500	hsa-miR-450b-5p	-2.22	99.34	v	v
*AP1S2*	9.40	hsa-miR-204-5p	-3.22	99.26	v	v
*PHF21A*	-6.38	hsa-miR-766-3p	2.35	99.73	v	v
*LPP*	-6.08	hsa-miR-141-3p	2.37	99.84	v	v
*LPP*	-6.08	hsa-miR-150-5p	5.61	99.66	x	v
*LPP*	-6.08	hsa-miR-193a-3p	2.00	99.89	x	v
*LPP*	-6.08	hsa-miR-3622a-5p	4.35	99.85	x	x
*LPP*	-6.08	hsa-miR-4792	2.21	99.03	x	x
*LPP*	-6.08	hsa-miR-6511b-3p	3.16	99.64	x	x
*LPP*	-6.08	hsa-miR-760	4.78	99.92	x	v
*TFAP2A*	3.81	hsa-miR-424-5p	-2.49	99.38	x	v

**Table 6:** Networks associated with 14 candidate genes differentially expressed in OA FLS

	Top Diseases and Functions	Score	Focus Molecules	Molecules in Network
1	Cellular Development, Cellular Growth and Proliferation, Neurological Disease	18	8	ARHGEF2, BMI1, CELF2, CITED2, CYR61, DCLK1, ETV1, FAP, ↑KIF1B, ↑LMO3, PRODH, ↓LPP, MELK, mir-224, mir-322, mir-515, ↑MRTFB(MKL2), MYC, NDRG1, NR2F2, NUP153, NUPR1, PAK3, ↓PHF21A, PLA2G16, POR, SAE1, ↑SENP2, SESN1, SHISA5, ↓SMAD4, ↑TFAP2A, TP53, TRIM33, ZMIZ1
2	Cell Cycle, Cell Death and Survival, Cellular Compromise	3	1	↑PTCD3, TNFRSF1A
3	Connective Tissue Development and Function, Cancer, Cell Cycle	3	1	↑AP1S2, SMARCA4
4	Cell Morphology, Cellular Assembly and Organization, Cellular Compromise	3	1	↑KCTD20, MARK4
5	Cardiac Arteriopathy, Cardiovascular Disease, Organismal Injury and Abnormalities	2	1	CACNA1C, ↑CACNA2D1, CACNB3
6	Cellular Development, Cellular Growth and Proliferation, Embryonic Development	2	1	FANCC, FYB1, GRB2, ↑SKAP2, SOX11

The genes marked in **bold** were the target genes identified in OA FLS.
